# The Clinical Value and Variation of Antithyroid Antibodies during Pregnancy

**DOI:** 10.1155/2020/8871951

**Published:** 2020-10-21

**Authors:** Chuyu Li, Jing Zhou, Zengshu Huang, Xinyao Pan, Wingting Leung, Lijia Chen, Yanzhi Zhang, Lan Wang, Yizhen Sima, Hans-Jürgen Gober, Na Zhang, Xuemin Qiu, Lisha Li, Liang Guan, Ling Wang

**Affiliations:** ^1^Laboratory for Reproductive Immunology, Hospital and Institute of Obstetrics and Gynecology, Shanghai Medical College, Fudan University, Shanghai, China; ^2^The Academy of Integrative Medicine of Fudan University, Shanghai, China; ^3^Shanghai Key Laboratory of Female Reproductive Endocrine-Related Diseases, Shanghai, China; ^4^Department of Pharmacy, Neuromed Campus, Kepler University Hospital, 4020 Linz, Austria; ^5^Department of Nuclear Medicine, Ruijin Hospital, School of Medicine, Shanghai Jiaotong University, Shanghai, China; ^6^Department of Nuclear Medicine, Ruijin Hospital North, School of Medicine, Shanghai Jiaotong University, Shanghai, China

## Abstract

Antithyroid antibodies, which include thyroid-stimulating hormone receptor antibodies (TRAbs), thyroid peroxidase antibodies (TPOAbs), and thyroid globulin antibodies (TgAbs), are widely known for their tight association with thyroid autoimmune diseases. The variation in all three kinds of antibodies also showed different trends during and after pregnancy (Weetman, 2010). This article reviewed the the physiological changes, while focusing on the variation of thyroid antibodies concentration in women during and after pregnancy, and adverse consequences related to their elevation. Since abnormal elevations of these antithyroid antibodies may lead to adverse outcomes in both mothers and fetuses, special attention must be paid to the titer of the antibodies during pregnancy. The molecular mechanisms of the variations in those antibodies have yet to be explained. The frequency and timing of thyroid antibody measurement, as well as different reference levels, also remain to be elucidated.

## 1. Introduction

Progesterone and androgens are considered to have immunosuppressive effects and are therefore protective in autoimmune diseases, and estrogen is generally considered to be immunostimulatory and therefore pathogenic in autoimmune diseases [1]. Due to the effects of estrogen on the immune system, women are more susceptible to autoimmune thyroid diseases (AITDs), which include Hashimoto's thyroiditis (HT), Graves' disease (GD), and idiopathic hypothyroidism [2]. Positivity for antithyroid antibodies are tightly associated with AITDs, and the diagnoses of GD and HT highlight the importance of measuring thyroid-stimulating hormone receptor antibodies (TRAbs), thyroid peroxidase antibodies (TPOAbs), and thyroid globulin antibodies (TgAbs). For GD, it is recommended to measure TRAbs before stopping antithyroid drug (ATD) treatment and during pregnancy for accurate diagnosis/differential diagnosis [3, 4]. TPOAbs and TgAbs are important indicators for determining the cause of primary hypothyroidism and the main indicators for diagnosing AITDs (including HT, atrophic thyroiditis, etc.) [5]. According to their effects on thyroid-stimulating hormone receptor (TSHR), TRAbs can be subdivided into TSHR-stimulating antibodies (TSAbs), TSHR-blocking antibodies (TBAbs), and neutral TRAbs (N-TRAbs), and these antibodies have different molecular structures and properties [6]. The abnormal elevation of TRAbs disturbs the thyroid function of both pregnant women and the fetus since they are capable of crossing the placental barrier freely [6]. Thyroid peroxidase (TPO), a membrane-bound enzyme, catalyzes the oxidation of iodide and the iodination of the tyrosyl residues of thyroglobulin. TPOAbs can bind to TPO and then damage the thyrocytes and cause hypothyroidism. The increase of TPOAbs is not scarce in the euthyroid population, and they are associated with major alterations in the course of pregnancy affecting the mother, fetus, and/or neonate [7]. Among the three kinds of antibodies, the TgAb is the earliest-discovered and it is mainly composed of IgG. It mainly attacks different antigenic determinants of thyroglobulin, which is located in the colloid of thyroid follicles. Since thyroglobulin is the intermediate of the thyroid hormones, TgAbs have a close association with both the synthesis and secretion of thyroid hormones [8]. TPOAb and TgAb are frequently present in the same individual. Some people think that the cross-reactivity between Tg and TPO plays a role in the origin of thyroid autoimmunity [9]. Such cross-reactivity could include either the epitopes recognized by autoantibodies and/or the epitopes recognized by T cells that provide ‘help' to B cells to differentiate and secrete antibodies [10]. Maternal thyroid disorders can damage maternal and fetal health during pregnancy, thus leading to adverse maternal, fetal, and obstetrical outcomes. Hypothyroidism is associated with gestational hypertension, anemia, preeclampsia, miscarriage, low fetal birth weight, fetal death, respiratory distress, congenital circulatory system malformations, and so on. Hyperthyroidism is associated with stillbirth, congestive heart failure, preterm labor, small for gestational age, and preeclampsia [11]. According to what we have described before, there is a strong connection among abnormal elevations of antithyroid antibodies, thyroid disease, and pregnancy.

It is generally considered that reduction in serum thyroid antibodies levels (TRAbs, TPOAbs, TgAbs) is the consequence of maternal immune tolerance during pregnancy. Relevant results have already been published. Researchers have found other major histocompatibility complex (MHC) molecules such as HLA-C, -E, -F, and HLA-G [12] instead of HLA class I and class II proteins to avoid allogeneic responses at the maternal-fetal interface. T-reg cells can also play a part in the depression of autoimmune responses in pregnancy. Estrogen has also been shown to be capable of dampening the function of B cells in pregnancy [13].

In this review, we will cover the interaction among them, especially between abnormal elevations of antithyroid antibodies and pregnancy.

## 2. Physiological Adjustment after Conception

### 2.1. Human Chorionic Gonadotropin (hCG)

**Figure 1:**
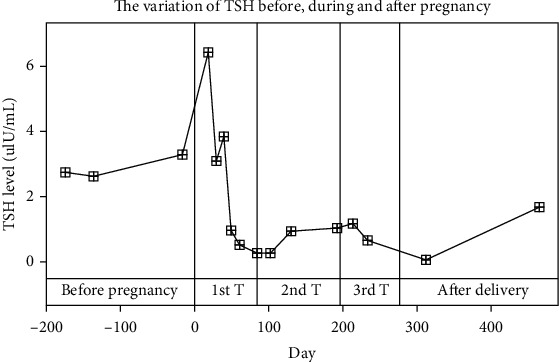
The variation in TSH before, during, and after pregnancy. The curve is a set of data from one patient at Ruijin Hospital. The delivery date of her infant was December 1, 2017. The last menstrual period was February 27, 2017, according to the gestational weeks and delivery date. The TSH level dropped to the lowest level at the end of the first trimester and then gradually ascended. T: trimester.

Increased hCG, which is produced by syncytiotrophoblasts, is one of the notable characteristics of early pregnancy. It can facilitate the synthesis as well as the secretion of thyroid hormones by stimulating TSHR, since the function of hCG is similar to the thyroid-stimulating hormone (TSH). The reason for the similarity is that the hCG and TSH molecules share similarities, as do the hCG and TSH receptors [14]. The level of hCG reaches its peak at the end of the first trimester, then decreases and remains stable during the second and third trimesters. The pattern of the change in free thyroxine (FT4) in the first trimester is the same as that of hCG. Because of the negative feedback effect of FT4, serum TSH in the first trimester (especially 7 to 12 weeks of gestation) drops to the lowest point and presents a mirror image of hCG peak [15–17]. One set of TSH data from a patient in Ruijin Hospital exhibited the same pattern as we described (Figure 1). The titer of serum TSH in 10-20% of normal pregnant women can be transiently subnormal, depending on the degree of the indirect suppression effect of circulating hCG [11].

### 2.2. Thyroxine-Binding Globulin (TBG)

TBG functions as the main carrier of thyroxine (T4) or triiodothyronine (T3). Total T4 (TT4) or total T3 (TT3) includes both the free forms (FT4 or free T3, FT3) and the TBG-bound hormones. FT4 and FT3 represent bioavailable hormones, whereas TT4 and TT3 represent serum hormones. Both estrogens and silylation lead to an increase in TBG because the clearance of the more common silylation form is reduced compared to that of the nonsilylation form [15]. As a result, the TBG serum level will double compared with the nonpregnancy level. Serum TT4 and TT3 will increase correspondingly, but by no more than 1.5 times the upper level of the reference range of the nonpregnant state [11]. The fetal serum TBG level will be 100 nmol/L (5 mg/L) at 12 weeks of gestation and will increase up to 500 nmol/L (25 mg/L) when the infant is born [18]. The progressive increase in fetal serum FT4 levels along with TT4 levels indicates the maturation of the hypothalamic-pituitary-thyroid axis [19].

### 2.3. Iodine Intake

Many factors can lead to the increase in iodine requirements associated with pregnancy [20]. The loss of iodide is mainly caused by the increased clearance from the kidney. On the other hand, an increased consumption of iodine is caused by the needs of the fetus, increased production of TBG, and placental deiodination of thyroid hormones. The WHO [21], Endocrine Society [20], and American Thyroid Association [22] recommend an intake of potassium iodide of approximately 250 mcg per day during pregnancy and lactation.

### 2.4. Thyroglobulin (TG)

TG is the precursor of T3 and T4 and is stored in the colloid of thyroid follicles. When the synthesis of thyroid hormones is activated, TG will be absorbed into thyroid follicular cells and processed into T3 and T4. The typical variation pattern of TG includes an increase in early pregnancy, a stable level throughout mid-pregnancy, and another increase at 36 weeks of gestation. The serum TG level returns to the same level as that of the nonpregnant state after delivery [23]. If there was an insufficient iodine supply, more TG would be released into the blood directly without being processed. Thus, some recent studies have attempted to use serum TG levels as predictors of iodine deficiency, with some issues remaining unsolved [24]. To make TG function a reliable tool to identify iodine deficiency in pregnant women, these issues must be overcome [25].

### 2.5. Volume of Thyroid Gland

The volume of the thyroid gland may be influenced by many factors, such as iodine supplementation, sex, TSH, parity, genetics, age, anthropometric parameters, and smoking [26]. In nonpregnant individuals, thyroid volume correlates positively with age, bodyweight, BMI, and total body water [27, 28]. In regard to pregnancy, BMI has been found to be positively correlated with thyroid volume [29]. The reason pregnancy is characterized by hypervolemia is that total body water will increase from 6 L to 8 L, and the extracellular water will also increase correspondingly since it composes the largest part of the total body water. Both body weight and BMI will increase with the accumulation of total water, and increase of body weight, BMI and total body water lead to an increase in thyroid volume [14]. The effect of TSH on thyroid volume in pregnant women is controversial [27, 29]. If the iodine supply is sufficient during pregnancy, the thyroid volume will not change significantly compared with that of the nonpregnant state [30]. Thyroid volume increases during pregnancy and decreases after delivery [29]. Based on these results, some researchers postulated that the increase in thyroid volume was caused by the increase in blood supply to the thyroid gland during pregnancy. This point of view is supported by the result of color flow Doppler sonography showing that intrathyroidal blood flow increases during pregnancy [31] and decreases in the year after delivery [29]. Thus, the authors hypothesized that the hemodynamic changes caused by delivery contributed to the changes in thyroid volume.

## 3. Clinical Significance of TRAbs, TPOAbs, and TgAbs

### 3.1. TRAbs

#### 3.1.1. Subclasses of TRAbs

TSHR belongs to the family of G protein-coupled receptors (GPCRs). When TSHR is activated, all four subfamilies of G proteins (Gs, Gi/o, Gq/11, and G12/13) can be recruited. TSHR-Abs are commonly oligoclonal and of the IgG1 subclass, although other isotypes have been reported [32, 33]. According to the different influences on TSHR (including “stimulating,” “blocking,” and “neutral”), TRAbs can be divided into TSAbs, TBAbs, and N-TRAbs [6, 34]. TSAbs are typical antibodies of GD [35]. When they bind to and activate TSHR, Gs and Gq/11 will be recruited [34]. The activation of G protein-coupled signaling pathways leads to subsequent reactions in thyroid epithelial cells, including depression of apoptosis, proliferation, and the excessive production and secretion of the thyroid hormone. Then, the invalidation of the regulation by TSH results in the elevation of serum thyroid hormones, which leads to the signs and symptoms of GD [36].

The main pathology of atrophic thyroiditis (AT) is the apoptosis of thyroid epithelial cells mediated by T cells [37]. In AT patients, TRAbs bind to TSHR without activating it and prevent the combination of TSH and TSHR, resulting in hypothyroidism. This kind of antibody is called TBAbs. Although GD is characterized by an increase in TSAbs, TBAbs may appear simultaneously and contribute to the fluctuation in thyroid function [36]. Moreover, the transformation from GD to hypothyroidism may occur in a small number of GD patients due to the transformation of TSAbs to TBAbs. TBAbs-induced hypothyroidism and HT are clinically indistinguishable, except for the positivity for TRAbs in the first type.

Both TSAbs and TBAbs prevent TSH from binding to thyroid epithelial cells through occupying TSHR. Thus, they are also called “TSH-binding inhibitory immunoglobulin (TBII)” [38]. Two main methods are currently used to measure TRAbs: “receptor assays” measuring TBII and “bioassays” measuring the capacity of TRAbs to stimulate (TSAbs) or depress (TBAbs) the production of cAMP mediated by their ability to combine with TSHR [39].

N-TRAbs do not block the binding of TSH to TSHR, but they are able to aggravate local infiltration of inflammatory cells in the thyroid or posterior orbit by promoting the production of mitochondrial reactive oxygen species (mROS), which then induces cellular apoptosis [34]. Both mROS and apoptosis are implicated in the pathogenesis of thyroid autoimmunity and in Graves' orbitopathy (GO), and GO is characterized by proptosis, eyelid retraction, lagophthalmos, pressure in the orbit and periorbital swelling.

#### 3.1.2. Clinical Application of TRAbs

As we have stated before, abnormal elevations of TPOAbs and TgAbs can be found in euthyroid populations. In contrast, abnormal increase of TRAbs can only be found in the sera of most GD patients and 10 to 15% of HT patients [36]. Their specificity for GD is close to 100%, and their sensitivity is approximately 80% in liquid-phase TRAb assays [40]; thus, the abnormal elevation of TRAbs is also considered the hallmark of GD. They can also be used to diagnose and predict the outcome of GO in some patients [40]. 80% of GD can be diagnosed by clinical examination, laboratory examination (FT4, FT3, and TSH), thyroid ultrasound, and thyroid scintigraphy. However, measurement of TRAbs cannot be replaced for some special characteristics. These characteristics include high sensitivity, high efficacy for obtaining information regarding proper therapeutic methods, and relatively low costs [40]. In addition to diagnosing GD and GO, the abnormal elevation of TRAbs can be used for the differential diagnosis of painless thyroiditis, unilateral exophthalmos, subclinical hyperthyroidism, euthyroid GO, and other ambiguous diseases [41]. The abnormal elevation of TRAbs may help distinguish type 1 (autoimmune) amiodarone-induced thyrotoxicosis from type 2 (inflammatory) [42]. After the application of antithyroid drugs (ATDs), the abnormal elevation of TRAbs can be used to predict GD recurrence risk [43]. TRAbs correlates positively also with thyroid vascularization at color-flow doppler and the odds of hyperthyroidism recurrence [44]. It has been reported that in GD patients developing hypothyroidism, TSAbs increase significantly 6 months after radioiodine treatment. Thus, the abnormal elevation of TSAbs is thought to be able to reflect thyroid damage caused by radioiodine and predict the outcome of thyroid function after radioiodine treatment [45]. Once basal serum TSH elevates abnormally and AITDs are considered, TBAbs may be the chief culprit, and the measurement of TBAbs may help establish the diagnosis of humoral immunity-induced hypothyroidism [46].

#### 3.1.3. Diseases Associated with the Abnormal Elevation of TRAbs in the Field of Obstetrics and Gynecology

Thyroid autoimmunity is relevant to infertility, miscarriage, preterm delivery, and postpartum depression [6, 47]. GD, the pathological basis of which is hyperthyroidism, has been shown to correlate with pregnancy. The undesirable pregnancy outcomes caused by hyperthyroidism are directly associated with the duration of thyrotoxicosis during pregnancy. Pregnancy-induced hypertension (PIH) accounts for the highest proportion of pregnancy complications caused by GD. Some researchers have found that the risk of eclampsia might increase by 5 times in hyperthyroid women without control compared to well-controlled women and nonhyperthyroid pregnant women [48, 49]. The combination of hypertension and left ventricular dysfunction triggered by prolonged thyrotoxicosis may proceed to congestive heart failure. In addition, many complications, such as still birth, intrauterine growth restriction, gestational diabetes mellitus (GDM), preterm birth, cesarean delivery, and low-birth weight infants, are associated with overt hyperthyroidism [50, 51]. The abnormal elevation of TRAbs may lead to poor growth and tachycardia of the fetus by crossing the placenta and stimulating the thyroid of the fetus [52]. Coincidentally, TBAbs may also cross the placenta and enter the circulation of the fetus, block the activation of TSHR, and induce neonatal transient hypothyroidism [36, 53]. If infants are diagnosed with congenital [54] or neonatal [55] hypothyroidism, the abnormal elevation of TBAbs would be detected in the serum of their mothers. Also, TBAbs can be found in the neonates from hypothyroid mothers [6].

### 3.2. TPOAbs

#### 3.2.1. Brief Introduction to TPOAbs

TPO functions as the core enzyme during the synthesis of thyroid hormones and is also the antigen of TPOAbs. The abnormal elevation of TPOAbs is most likely to be detected at the onset of thyroid dysfunction. Both TPOAbs and TgAbs mainly belong to the immunoglobulin (Ig) G class, mainly IgG1, with some IgG2, little IgG3, and tiny IgG4 subclasses. IgA class TPOAbs can be found in AITD patients at much lower levels [10, 33]. They are produced by lymphocytes that infiltrate the thyroid, and the titer has a positive correlation with the severity of infiltration [56]. Many pathological effects of the abnormal elevation of TPOAbs have been discovered by researchers. TPOAbs bind to TPO to form an immune complex (IC), which will trigger subsequent reactions, including antibody-dependent cell-mediated cytotoxicity (ADCC) and complement-mediated cytotoxicity, and then destroy thyroid epithelial cells [8]. Some studies have reported that TPOAbs were positive in 10 to 15% of the healthy population, and clinical hypothyroidism may develop from subclinical hypothyroidism in 2% of the healthy population. The TPOAb-positive rate varies with age, race, and ethnic background [57], and it can be higher in women of reproductive age. One study found that this rate ranged between 5.4% and 20% among those women, and it reached 14 to 33% in women with recurrent miscarriages and infertility [58].

Some factors may influence serum TPOAb titer. The effect of selenium to TPOAbs is controversial. Some scientists have found the use of selenium may lead to the drop in the titer of TPOAb [59–63], but other researchers have different opinions [64]. The effect of amiodarone on thyroid autoimmunity is still unclear [65]. Treatment of 131I or interferon-*α* will lead to the increase of TPOAbs [10, 66]. The review of Rayman has mentioned that appearances of the abnormal elevation of TPOAbs and TgAbs are common in populations with a stable high iodine intake and those with mild and moderate iodine deficiency [67]. According to Snijders et al., in bipolar disorder (BD) patients, the prevalence of TPO-abs was unrelated to lithium use [68].

#### 3.2.2. Clinical Application of TPOAbs

The abnormal elevation of the TPOAb titer is the gold standard for diagnosing HT and an assistant index for diagnosing GD. This index is sensitive and precise and prevails against fine-needle aspiration cytology of the thyroid when diagnosing HT [8]. TPOAb titer positively correlates with the TSH level, and it precedes the occurrence of thyroid failure. Thus, the abnormal elevation of TPOAbs is thought to be the marker of early thyroid dysfunction. TPOAbs are also considered to be able to reflect the severity of lymphocyte infiltration, and this ability is independent of hypothyroidism. TPOAb positivity is one of the risk factors for hypothyroidism in special conditions such as IFN-*α*, IL-2, lithium, and amiodarone treatment and in Down's syndrome patients [56].

#### 3.2.3. Diseases Associated with the Abnormal Elevation of TPOAbs in the Field of Obstetrics and Gynecology

TPOAb-positive women will have an increased risk of hypothyroidism if they were pregnant [56]. The risk of hypothyroidism and subclinical hypothyroidism will increase by 2-fold after pregnancy if an individual is TPOAb-positive before pregnancy [69]. The appearance of the abnormal elevation of TPOAbs is also a risk factor for other medical diseases such as GDM [69] and anemia [70] during pregnancy. The relationship of the abnormal elevation of TPOAbs with obstetric complications is also nonnegligible since many studies have been reported by scientists. Associated complications include miscarriage, premature delivery, perinatal death, low delivery rate, polyhydramnios and placental abruption, and the abnormal elevation of TPOAbs are a risk factor for all of these complications [7, 69–77]. The presence of the abnormal elevation of TPOAbs increases the rate of postpartum thyroid dysfunction (PPTD) [56], postpartum thyroiditis (PPT) [75], and postpartum depressive symptoms [78–80]. One recent study found a higher prevalence of infertility in TPOAb-positive euthyroid females [70]. However, some researchers have different opinions, and their study only revealed the association between premature rupture of fetal membrane (PROM), low birth weight, and the abnormal elevation of TPOAbs [81]. Other studies by Łukaszuk et al. and Plowden et al. did not find differences in fertilization, pregnancy rates, live birth rates, implantation, or miscarriage rates between TPOAb-positive and TPOAb-negative patients [82, 83].

It is worth mentioning that the fetus can also be influenced by the maternal abnormal elevation of TPOAbs. In the first 18 to 20 weeks of pregnancy, high concentrations of hCG will stimulate the thyroid gland to ensure that the developing fetus has sufficient thyroid hormone utilization [84]. However, TPOAb-positive women have impaired thyroid response to hCG stimulation [84]. Derakhshan et al. found that maternal TPOAb positivity was associated with lower mean child IQ in Rotterdam patients [84]. Findings of Ghassabian et al. imply that the elevated titers of TPOAbs during pregnancy impact increase risk of problem behavior in children, in particular, attention deficit/hyperactivity [85]. The study of Pop et al. showed that children of pregnant women who had elevated titers of TPOAb but normal thyroid function are at risk for impaired development, including diminished verbal, perceptual, cognitive, and motor performance [86]. Wasserman et al. found that antenatal exposure to maternal TPOaAb during the third trimester of pregnancy is associated with impaired auditory development [87]. The relevance between the abnormal elevation of TPOAbs and offspring autism has also been reported by researcher [88]. The abnormal elevation of TPOAbs has not been reported as a causal factor of parity, period of gestation, or body weight [71].

### 3.3. TgAbs

#### 3.3.1. Clinical Application of TgAbs

If autoimmune abnormalities occur in the thyroid, the abnormal elevation of TgAbs frequently appears along with that of TPOAbs [8]; thus, TgAbs are also regarded as markers of AITDs. Both TgAbs and TPOAbs are detected when the patient is suspected to have autoimmune hypothyroidism. However, TgAb alone in the absence of TPOAb is not significantly associated with thyroid disease [10, 57, 89]. In addition, TPOAb are more prevalent than TgAb in GD [10]. The sensitivity and specificity of TgAbs are not as good as those of TPOAbs in the diagnosis and prognosis of AITDs [5, 8, 10]. All of these factors may weaken the necessity of the measurement for TgAbs since the test for TPOAbs alone seems enough. Nevertheless, in the view of McLachlan et al., there are three reasons for measuring TgAb: Thyroid cancer, clinical versus subclinical disease, and insight into disease pathogenesis [10]. Women with isolated TgAb had significantly higher serum TSH levels compared to those in women without thyroid autoimmunity [90]. Thus according to “2014 European Thyroid Association Guidelines for the Management of Subclinical Hypothyroidism in Pregnancy and in Children”, in the case of elevated TSH and negative TPOAb, TgAb should be measured [91]. The appearance of the abnormal elevation of TPOAbs and their powerful supplement TgAbs indicates the severity of thyroid lesions [8].

#### 3.3.2. Diseases Associated with the Abnormal Elevation of TgAbs in the Field of Obstetrics and Gynecology

One study found that the abnormal elevation of titers of both TPOAbs and TgAbs in the first trimester of pregnancy were linked to an increase in morbidity due to hypertensive disorders of pregnancy, which was mainly caused by an increased risk of gestational hypertension, and the complications had no relationship with thyroid function. However, an association between the abnormal elevation of TgAbs and the risk of gestational hypertension or preeclampsia in the second trimester was not found [92]. Their results have been supported by another study that included 5805 women from 12 to 20 weeks of gestation, and the study did not find an association between abnormal elevations of thyroid antibodies (TPOAbs or TgAbs) and preeclampsia [93]. One study by Ordookhani et al. reported a positive correlation between transient neonatal hypothyroidism and elevated serum TgAb levels [94].

## 4. The Variation in TRAbs, TPOAbs, and TgAbs during and after Pregnancy

### 4.1. TRAbs

#### 4.1.1. TRAb Trend during and after Pregnancy

The concentration of TRAb tends to decrease throughout pregnancy and increases after delivery. However, sometimes pregnant women may have stable or unexpectedly increased titers during pregnancy [95]. According to the pattern of TRAb change, it is thought that the decrease in TRAbs throughout pregnancy is caused by pregnancy-induced immunosuppression [6].

In the study of Gonzalez-Jimenez, 15 healthy and 45 pregnant GD women (20 were in recovery and 20 were well-managed with ATDs during pregnancy) were recruited. At first TRAb titers were in the normal range, but tended to decrease during late gestation. Even though TRAb titers of most individuals remained within the normal range, a significant rebound was observed in the late postpartum period [96]. In a study from Japan, researchers used four methods (first-, second-, and third-generation TBII assays and a bioassay) to measure the serum TRAb level in 23 women during pregnancy. They observed a decrease in both TRAbs and TSAbs during the pregnancy, independent of the assay method used [97]. A study by Abeillon-du Payrat et al. obtained the result of TBII from 42 pregnant women; among which, the levels decreased or remained stable in 36 women and the levels increased unexpectedly in the remaining six women [39]. A case report by Yoshida et al. observed that during the second pregnancy, the titer of serum TBAb and TRAb showed a remarkable fall, and their activities became undetectable after 36 weeks of gestation. One month after the second delivery, TBAb level increased with a concomitant rise of serum TRAb level [98]. The study of Amino et al. included six GD patients, and they found that activities of TSAbs and TBAbs decreased during pregnancy and increased after delivery [99]. Balucan et al. confirmed that the rebound of the TSAb level was most likely to occur four to twelve weeks postpartum. The rebound of the TSAb level might lead to the recurrence of or new-onset GD [66]. However, different results have occurred in some pregnant women. TRAbs can remain stable throughout pregnancy regardless of the incipient level since the course of GD is variable regardless of pregnancy [6].

#### 4.1.2. The Switch of the Subclasses of TRAbs

The “quantitative” change in TRAbs has been observed as we have elaborated before. The “qualitative” change, or the variation in subclasses of TRAbs, has also been shown in both pregnant and nonpregnant states. Patients treated with levothyroxine (LT4) may confront the switch from TBAbs to TSAbs, while the reverse switch occurs in patients treated with ATDs [100]. The switch between the two kinds of antibodies may lead to the remission and deterioration of thyrotoxicosis in pregnancy. In a study that recruited fifteen pregnant GD women with low-dose ATD management and fourteen healthy pregnant women, serum levels of TRAbs (assessed by receptor assays), TSAbs, and TBAbs (by bioassays) were tested throughout pregnancy. All antibodies were negative in the healthy pregnant women, while TSAbs decreased significantly and TBAbs increased significantly with the fluctuation of TRAbs (TBII) in pregnant GD women [101]. Kung et al. recruited thirteen pregnant women with GD, and five of them without antithyroid management (patients 3, 5, 8, 10, and 13) were TSAb-active in the first trimester. The level of TSAbs decreased in four patients, except patient 8, as pregnancy progressed. For TBAbs, three patients (patients 4, 5, and 9) were TBAb-positive in the first trimester. By the second and third trimesters, the number of TBAb-positive patients increased to eight and nine, respectively. TBAbs in seven patients remained positive four months after delivery [102]. The switch may also occur after delivery. The case report by Yoshida et al. showed the transformation from TBAbs to TSAbs after eight months postpartum [98].

#### 4.1.3. The Significance of TRAb Monitoring

Pregnancy outcomes depend on metabolic status at delivery. If pregnant women with thyroid disorders are treated properly from the beginning of pregnancy and maintain euthyroid, they may have better pregnancy outcomes [103]. However, TRAb levels may change after the main treatment of GD (including thyroidectomy, ATDs, and I131 treatment) [104–107], and TRAb-positive euthyroid women do exist [41]. Even if the thyroid function indexes of pregnant women are normal, the fluctuation of TRAbs must be monitored since thyroid function examinations alone are not able to predict the effect of the abnormal elevation of TRAbs on both the mother and fetus. TRAbs can also be used to distinguish between the syndrome of gestational hyperthyroidism (SGH) and GD [108]. A maternal TRAb serum concentration >5 IU/L (approximately 3 times the upper limit of normal for the assay) in the second and third trimester predicted neonatal hyperthyroidism [39, 109].

As for fetuses and infants, the study by Zakarija et al. reported that in one pregnant GD woman, serum TSAbs were lower during pregnancy than the levels in paired postpartum samples, but the infants were hyperthyroid after delivery. Therefore, they thought pregnancy might depress TSAbs; but once the effect was not sufficient, the influence of the abnormal elevation of TSAbs on the neonate's thyroid could not be avoided [110]. Researchers have proposed measuring TRAb levels during the third trimester of pregnancy to predict the risk of neonatal thyroid dysfunction [111].

The importance of monitoring TRAbs after delivery cannot be neglected either. TSAbs can be used as a predictor of postpartum onset or recurrence of GD since the elevation of TSAbs precedes the onset of the symptoms of GD when measured by means of sensitive bioassays [112]. Measurement of TRAbs postpartum can also be used to distinguish GD from postpartum thyroiditis [40].

### 4.2. TPOAbs and TgAbs

#### 4.2.1. The Variation in TPOAbs and TgAbs during and after Pregnancy

**Figure 2:**
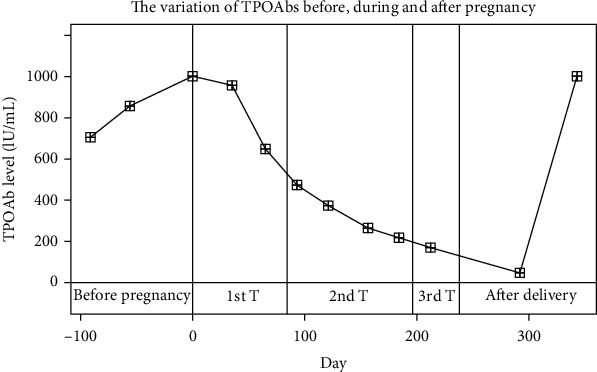
The variation in TPOAbs before, during, and after pregnancy. The curve is a set of data from one patient at Ruijin Hospital. The patient suffered from Hashimoto's thyroiditis, and the delivery date of her infant was July 2, 2018. The last menstrual period was November 6, 2017, according to the gestational weeks and delivery date. TPOAb levels decreased during pregnancy and rebounded after delivery. T: trimester.

**Figure 3:**
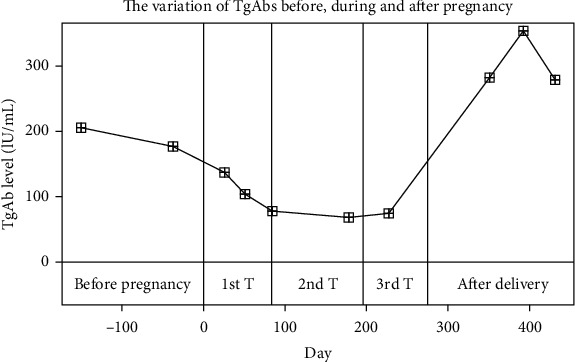
The variation in TgAbs before, during, and after pregnancy. The curve is a set of data from one patient at Ruijin Hospital. The delivery date of her infant was July 12, 2017. The last menstrual period was October 10, 2016, according to the gestational weeks and delivery date. The TgAb level decreased during pregnancy and rebounded after delivery. T: trimester.

The variation of TPOAbs and TgAbs over time are similar to those of TRAbs. Many researchers have observed that serum levels of TPOAbs and TgAbs gradually decrease during pregnancy but rebound after delivery [92, 113–116]. Their findings are the same as those we observed in the patients in Ruijin hospital (Figures 2 and 3). Smyth et al. recruited 25 pregnant women as the observation group and 57 nonpregnant women as the control group. They found that the TPOAb-positive rates of the control group and the rates in the first, second, and third trimesters of the observation group were 26.3%, 8.0%, 4.0%, and 0%, respectively [117]. In a recent study including forty TPOAb-positive euthyroid women, the average serum TPOAb levels were 209 ± 284.32 IU/ml, 112.34 ± 126.08 IU/ml, 76.06 ± 83.44 IU/ml, and 84.13 ± 106.32 IU/ml in the first, second, and third trimester in pregnancy and postpartum period, respectively. The TPOAb titer decreased significantly in the second and third trimesters compared to that in the first trimester (*p* = 0.016 and 0.003, respectively) [70]. Smyth et al. found that TgAb-positive rates were 36.8%, 12.0%, 4.0%, and 0% in the nonpregnant group and in the first, second, and third trimesters of the pregnancy group, respectively. The TgAb-positive rate of the pregnant group six weeks postpartum did not change compared to that of the third trimester (0%) [117].

Some studies have focused on the postpartum period. Chen et al. allocated 26 women into a TPOAb-positive group and 182 women into a TPOAb-negative group. The postpartum TPOAb level increased significantly compared with the antepartum level in the TPOAb-positive group, but this result was not observed in the TPOAb-negative group [75]. Feldt-Rasmussen et al. found that TPOAbs increased six months postpartum in 36 TgAb-positive and TPOAb-positive mothers, and the increase was independent of PPTD [118]. According to the study of Smyth et al., the prevalence of TPOAbs was 21.7% at six weeks postpartum compared to 0% in the third trimester [117]. According to a study by Parkes et al., the average TPOAb level increased from 20 KIU/L to 121 KIU/L throughout the postpartum year in the euthyroid TPOAb-positive group. In the PPT group, the TPOAb level reached the peak level of 181 KIU/L five months postpartum, compared to 28 KIU/L at delivery, and dropped to 118 KIU/L by twelve months postpartum [119].

#### 4.2.2. The Significance of the Monitoring of TPOAbs and TgAbs

As we have stated before, the abnormal elevation of TPOAbs correlates with thyroid autoimmune disorders, and most of the patients are euthyroid [57, 73]. In addition, the presence of the abnormal elevation of TPOAbs precedes the onset of thyroid dysfunction in adults [120]. Researchers have found that the elevation of TSH by the last trimester of pregnancy might occur in 15 to 20% of TPOAb-positive euthyroid women [121]. The monitoring of TPOAbs in euthyroid subjects may help predict the occurrence of hypothyroidism. TPOAbs increase the risk of hypothyroidism even if the TSH level is within the normal range [120]. Measuring TPOAbs can be beneficial for women preparing for pregnancy and pregnant women [56]. Testing for TgAbs is also helpful, as we explained in part two. Monitoring TPOAbs and TgAbs also helps to detect postpartum diseases. The presence of the abnormal elevation of TPOAbs in early pregnancy is regarded as the most powerful marker for predicting PPTD [80, 122]. TPOAbs are also used as a predictor of postpartum depression [79]. Even after LT4 treatment, the TPOAb level was negative in a few patients by 50 months postpartum [123]. It is still controversial whether the levels of TPOAbs and TgAbs will drop after the recovery of thyroid function in HT patients treated with LT4 [123–127]. For fetuses and infants, we have stated that abnormal elevation of TPOAbs is associated with neurodevelopmental defects; thus, it's necessary that attention is given to maternal thyroid function and TPOAb titer to prevent neurodevelopmental defects, which include lower IQ, learning difficulties, hearing deficits, and cerebral palsy [128–130].

## 5. Conclusions

According to what we have mentioned before, during pregnancy, many endocrinal physiological changes may occur. The abnormal elevations of TRAbs, TPOAbs and TgAbs may lead to certain adverse outcome to mothers and/or fetus. The most common change patterns of the three kinds of antibodies are that they decrease during pregnancy and increase again after delivery. The importance of the monitoring of the three antibodies is also reflected in both Chinese and American guidelines for the management of thyroid dysfunction during pregnancy and the postpartum period, and relevant clauses have been listed for the clinical practitioner's reference (Table 1) [22, 108]. In addition, the diseases associated with the three antibodies that have been reported by other researchers are also included to emphasize the clinical value of those antibodies. Since thyroid antibodies do not necessarily imply that positive-women have thyroid dysfunction, therefore, thyroid antibodies should be measured along with thyroid function tests.

The diseases associated with TRAbs, TPOAbs, and TgAbs in the field of obstetrics and gynecology are listed in Table 1. Clauses relevant to those antibodies are cited from Guidelines of the American Thyroid Association for the diagnosis and management of thyroid disease during pregnancy and the postpartum period and Guidelines for the diagnosis and treatment of thyroid disease during pregnancy and the postpartum period (version 2012). *Abbreviations*: TRAbs: thyroid-stimulating hormone receptor antibodies; TPOAbs: thyroid peroxidase antibodies; TgAbs: thyroid globulin antibodies; GD: Graves' disease; GO: Graves' ophthalmopathy; PIH: pregnancy-induced hypertension; GDM: gestational diabetes mellitus; TSH: thyroid-stimulating hormone; FT4: free thyroxine; TT4: total thyroxine; SGH: syndrome of gestational hyperthyroidism; ATDs: antithyroid drugs; MMI: methimazole; PTU: propylthiouracil; HT: Hashimoto thyroiditis; PPTD: postpartum thyroid dysfunction; PPT: postpartum thyroiditis; IQ: intelligence quotient; LT4: levothyroxine; ART: assisted reproductive technology; AITDs: autoimmune thyroid diseases.

## Figures and Tables

**Table 1:** The diseases and guidelines associated with TRAbs, TPOAbs, and TgAbs.

	Diseases associated with antibodies	Relevant guidelines cited from the 2012 Guidelines of the Chinese Society of Endocrinology and Chinese Society of perinatal medicine for the diagnosis and management of thyroid disease during pregnancy and the postpartum period	Relevant guidelines cited from the 2017 Guidelines of the American Thyroid Association for the diagnosis and management of thyroid disease during pregnancy and the postpartum period
TRAbs	GD, GO, humoral immunity-induced hypothyroidism, infertility, miscarriage, preterm delivery, postpartum depression, PIH, left ventricular dysfunction, congestive heart failure, still birth, intrauterine growth restriction, GDM, preterm birth, and cesarean delivery Low birth weight, poor growth, tachycardia, goiter, hyperthyroidism, and neonatal hypothyroidism	TSH titer < 0.1 mIU/L implied the possibility of thyrotoxicosis, and the titer of FT4, TT4, TRAbs, and TPOAbs should be measured (recommendation rank A). SGH needs to be distinguished from GD, which is accompanied by oculopathy and positive TRAbs and TPOAbs. Thyroidectomy is the best choice in patients planning to conceive in 2 years with high TRAb titer. The dosage of ATDs can be reduced in the middle or late trimester of pregnancy of GD patients, 20 to 30% who can stop the use of ATDs. However, pregnant patients with a high level of TRAbs should be excluded and they should be treated with ATDs until the end of pregnancy. TRAb titer should be tested during thyroidectomy in order to assess the potential possibility of the occurrence of hyperthyroidism in the fetus. Indications for the monitoring of TRAbs in GD patients are active maternal hyperthyroidism, medical history of I131 treatment, medical history of delivery of hyperthyroid infants, medical history of thyroidectomy as the treatment for hyperthyroidism during pregnancy. Testing the TRAb titer is helpful to assess the pregnancy outcome. If the TRAb level is higher than three times than that of the reference level, the fetus should be monitored closely and with the involvement of a mother-infant therapist is recommended. The measurement of TRAbs between 20 and 24 weeks of pregnancy in GD patients or patients who had GD before is recommended, because the titer of TRAbs will help doctors assess the outcome of pregnancy (recommendation rank B). Monitoring fetal heart rate and fetal thyroid volume is recommended beginning in the middle period of pregnancy in pregnant women with high TRAb level (recommendation rank I).	Women with GD effectively treated with I131 ablation or surgical resection require TRAb monitoring beyond measurement of maternal thyroid function (strong recommendation, moderate-quality evidence). Measurement of TRAbs and maternal TT3 may prove helpful in clarifying the etiology of thyrotoxicosis (strong recommendation, moderate-quality evidence). In a newly pregnant woman with GD, who is euthyroid on a low dose of MMI (£5–10 mg/d) or PTU (£100– 200 mg/d), the physician should consider discontinuing all antithyroid medication given potential teratogenic effects. The decision to stop medication should take into account the disease history, goiter size, duration of therapy, the results of recent thyroid function tests, TRAb measurement, and other clinical factors (weak recommendation, low-quality evidence). If maternal TRAb concentration is high (>3 times the upper reference for the assay), the fetus should be carefully monitored for the development of fetal hyperthyroidism throughout pregnancy, even if the mother is euthyroid post thyroidectomy (strong recommendation, high-quality evidence). (a) If the patient has a past history of GD treated with ablation (radioiodine or surgery), a maternal serum determination of TRAbs is recommended during initial thyroid function testing during early pregnancy (strong recommendation, moderate-quality evidence). (b) If maternal TRAb concentration is elevated in early pregnancy, repeat testing should occur at weeks 18–22 (strong recommendation, moderate-quality evidence). (c) If maternal TRAbs are undetectable or low in early pregnancy, no further TRAb testing is needed (weak recommendation, moderate-quality evidence). (d) If a patient is taking ATDs for treatment of Graves' hyperthyroidism when pregnancy is confirmed, a maternal serum determination of TRAbs is recommended (weak recommendation, moderate-quality evidence). (e) If the patient requires treatment with ATDs for GD through midpregnancy, a repeat determination of TRAbs is again recommended at weeks 18–22 (strong recommendation, moderate-quality evidence). (f) If elevated TRAbs are detected at weeks 18–22 or the mother is taking ATD in the third trimester, a TRAb measurement should again be performed in late pregnancy (weeks 30–34) to evaluate the need for neonatal and postnatal monitoring (strong recommendation, high-quality evidence). Fetal surveillance should be performed in women who have uncontrolled hyperthyroidism in the second half of pregnancy and in women with a high TRAb level detected at any time during pregnancy (greater than 3 times the upper limit of normal). A consultation with an experienced obstetrician or maternal–fetal medicine specialist is recommended. Monitoring may include ultrasound to assess heart rate, growth, amniotic fluid volume, and the presence of fetal goiter (strong recommendation, moderate-quality evidence). A history of maternal thyroid illness, use of antithyroid medications (PTU, MMI) during gestation, or measurements of abnormal maternal thyroid function or TRAbs during gestation should be communicated to the newborn's neonatologist or pediatrician (strong recommendation, moderate-quality evidence).

TPOAbs	Overt hypothyroidism, subclinical hypothyroidism, latent hypothyroidism GD, HT, GDM, anemia, miscarriage, premature delivery, perinatal death, low delivery rate, polyhydramnios, placental abruption, PPTD, PPT, postpartum depression, and infertility Respiratory distress, neurodevelopmental deficits, low IQ, learning difficulties, and hearing deficits	The guideline does not support or oppose LT4 treatment in subclinical hypothyroid pregnant women with negative TPOAbs (recommendation rank I). Hypothyroid patients accompanied with positive TPOAbs during pregnancy should be treated with LT4 (recommendation rank B). The diagnostic criterion of positive thyroid autoantibodies is a TPOAb titer that surpasses the upper limit of the provided kit (recommendation rank A). The TSH titer of euthyroid pregnant women with positive TPOAbs should be checked every 4 to 6 weeks in the first half of pregnancy. During 26 to 32 weeks of pregnancy, the TSH titer should be detected at least once. Once TSH surpasses the normal range, LT4 treatment should be implemented (recommendation rank B). Treatment with LT4 cannot prevent the occurrence of PPT in TPOAb-positive pregnant women. The screening of TSH, FT4, and TPOAbs before the eighth week of pregnancy is recommended (recommendation rank B).	When possible, population-based trimester-specific reference ranges for serum TSH should be defined through assessment of local population data representative of a health care provider's practice. Reference range determinations should only include pregnant women with no known thyroid disease, optimal iodine intake, and negative TPOAb status (strong recommendation, moderate-quality evidence). Euthyroid pregnant women who are TPOAb-positive or TgAb-positive should have the measurement of serum TSH concentration performed at time of pregnancy confirmation and every 4 weeks through midpregnancy (strong recommendation high-quality evidence). Selenium supplementation is not recommended for the treatment of TPOAb-positive women during pregnancy (weak recommendation, moderate-quality evidence). Insufficient evidence exists to conclusively determine whether LT4 therapy decreases pregnancy loss risk in TPOAb-positive euthyroid women who are newly pregnant. However, administration of LT4 to TPOAb-positive euthyroid pregnant women with a prior history of loss may be considered given its potential benefits in comparison with its minimal risk. In such cases, 25–50 lg of LT4 is a typical starting dose (weak recommendation, low-quality evidence). Insufficient evidence exists to determine whether LT4 therapy improves the success of pregnancy following ART in TPOAb-positive euthyroid women. However, administration of LT4 to TPOAb-positive euthyroid women undergoing ART may be considered given its potential benefits in comparison to its minimal risk. In such cases, 25–50 lg of LT4 is a typical starting dose (weak recommendation, low-quality evidence). When available, population- and trimester-specific reference ranges for serum TSH during pregnancy should be defined by a provider's institute or laboratory and should represent the typical population for whom care is provided. Reference ranges should be defined in healthy TPOAb-negative pregnant women with optimal iodine intake and without thyroid illness (strong recommendation, high-quality evidence). Pregnant women with TSH concentrations > 2.5 mU/L should be evaluated for TPOAb status. Subclinical hypothyroidism in pregnancy should be approached as follows: (a) LT4 therapy is recommended for (i) TPOAb-positive women with a TSH greater than the pregnancy-specific reference range (see recommendation 1) (strong recommendation, moderate-quality evidence). (ii) TPOAb-negative women with a TSH greater than 10.0 mU/L (strong recommendation, low-quality evidence). (b) LT4 therapy may be considered for (i) TPOAb-positive women with TSH concentrations > 2.5 mU/L and below the upper limit of the pregnancy-specific reference range (weak recommendation, moderate-quality evidence). (ii) TPOAb-negative women and TPOAb-negative women with TSH concentrations greater than the pregnancy-specific reference range and below 10.0 mU/L (weak recommendation, low-quality evidence). (c) LT4 therapy is not recommended for - TPOAb-negative women with a normal TSH (TSH within the pregnancy-specific reference range or <4.0 mU/L if unavailable) (strong recommendation, high-quality evidence). Women with overt and subclinical hypothyroidism (treated or untreated) or those at risk for hypothyroidism (e.g., patients who are euthyroid but TPOAb-positive or TgAb-positive, posthemithyroidectomy, or treated with radioactive iodine) should be monitored with a serum TSH measurement approximately every 4 weeks until midgestation and at least once near 30 weeks gestation (strong recommendation, high-quality evidence). There is insufficient evidence to recommend for or against universal screening for abnormal TSH concentrations preconception, with the exception of women planning assisted reproduction or those known to have TPOAb positivity (no recommendation, insufficient evidence).

TgAbs	AITDs, gestational hypertension, and neonatal hypothyroidism		

## Data Availability

The data used to support the findings of this study are available from the corresponding author upon request.
